# Preliminary Announcement

**Published:** 1906-08

**Authors:** 


PRELIMINARY ANNOUNCEMENT.

The Monarch Book Company, Chicago, announce that they will shortly issue a California story by a native son, "Poker Jim, Gentleman," by G. Frank Lydston, M.D.

This is a story of the old-time California mining camps by a native son of the Golden West. It depicts in graphic terms the oddities and heroisms of the early Western pioneers and brings out in bold relief the many-sidedness of their characters. The delineation of these characters is a labor of love on the part of the author, who recalls them from his boyhood's days, and as the most picturesque features of his early life. They indeed belong to "the days when there were giants in the land."

---

### INTOXICATION WITH BETA EUCAIN.

Kraus (*Deutsche Med. Woch.*) relates that a patient requiring internal urethrotomy was injected beforehand with 10 c.c. of a 2 per cent. solution of beta eucain. He was a robust, phlegmatic man of forty. The injection was repeated the next day when he came to have the cut stricture stretched. Almost at once he felt faint and became partly unconscious, with increasing dyspnea, distress and chills, while the breathing grew shallower and shallower. The condition lasted in all for an hour or an hour and a half. The patient was able to eat then, but did not sleep much that night and found that he was somewhat forgetful for a day or so afterward. There was no actual collapse. Treatment was with wine and camphor, faradization of the phrenic nerves in the neck and inhalation of oxygen. Kraus remarks that it is the first time that any mishap has been observed from the use of beta eucain at the clinic (Lewin's Urologic Polyclinic), although it is in daily use there. He refers casually to the two cases of intoxication from beta eucain reported by Marcinowski. In one case the patient had a transient syncope, but he says that she was subject to fainting. In the other case, after injection of 1.5 c.c. of a 10 per cent. solution the patient exhibited tonic and clonic spasms, which gradually subsided after fifteen minutes, but were followed by stupor and weakness, the symptoms all passing away by the end of six hours. Dolbeau, he adds, has reported a similar case of intoxication after intravenous injection of beta eucain, and Simon has mentioned transient headache and vomiting in a man whose bladder had been filled with 80 c.c. of a 4 per cent. solution for a Bottini operation.—*Journal A. M. A., March 10.*

